# Interactive online calculator for estimation of muscle and hepatic insulin sensitivity in adults with Type 1 diabetes using clinical and research biomarkers

**DOI:** 10.1111/dom.70517

**Published:** 2026-02-02

**Authors:** Andrzej S. Januszewski, Jennifer R. Snaith, Greg M. Kowalski, Clinton R. Bruce, D. Jane Holmes‐Walker, Alicia J. Jenkins, Jerry R. Greenfield

**Affiliations:** ^1^ Sydney Pharmacy School University of Sydney Camperdown New South Wales Australia; ^2^ NHMRC Clinical Trials Centre University of Sydney Camperdown New South Wales Australia; ^3^ Department of Medicine University of Melbourne Fitzroy Victoria Australia; ^4^ Diabetes and Metabolism Garvan Institute of Medical Research Darlinghurst New South Wales Australia; ^5^ St Vincent's Clinical Campus University of New South Wales Darlinghurst New South Wales Australia; ^6^ Department of Diabetes and Endocrinology St Vincent's Hospital Sydney Darlinghurst New South Wales Australia; ^7^ Institute for Physical Activity and Nutrition, School of Medicine Deakin University Geelong Victoria Australia; ^8^ Institute for Physical Activity and Nutrition, School of Exercise and Nutrition Science Deakin University Geelong Victoria Australia; ^9^ Department of Diabetes and Endocrinology Westmead Hospital Westmead New South Wales Australia; ^10^ Sydney Medical School University of Sydney Camperdown New South Wales Australia; ^11^ Baker Heart and Diabetes Institute Melbourne Victoria Australia

## Abstract

**Aims:**

Impaired insulin sensitivity is an under‐recognised risk in Type 1 diabetes but is challenging to measure with ‘gold‐standard’ euglycaemic clamps. Adding stable‐isotope glucose distinguishes hepatic and muscle insulin action (assessed by endogenous glucose production [EGP] and glucose infusion rate [GIR], respectively). We therefore searched for a blood‐biomarker alternative.

**Methods:**

Two‐step clamps were conducted in 40 adults with Type 1 diabetes, participating in the INTIMET trial (INsulin resistance in Type 1 diabetes managed with METformin, ACTRN12619001440112). Participants were characterised with 33 baseline biomarkers.

**Results:**

Exhaustive search analyses derived a formula predicting an ‘unfavourable GIR’ (dichotomous variable: below median of 60.4 μmol/kg fat‐free mass [FFM]/min) using: total daily insulin dose (TDI), fasting triglycerides (TGs), insulin‐like growth factor 1 and aspartate aminotransferase levels (area under the receiver operating characteristic curve (AUROC) 0.97, *p* < 0.0001, *R* ^2^ [Nagelkerke] = 0.83, 92.5% accuracy). An ‘unfavourable EGP level’ (above median of 6.2 μmol/kg FFM/min) during low‐dose clamp was predicted by TDI, TGs, alkaline phosphatase and uric acid levels (AUROC 0.86, *p* = 0.001, *R* ^2^ (Nagelkerke) = 0.50, 80% accuracy). A free online tool (https://bit.ly/EGP-GIR-calculator) converts these variables into dichotomised EGP and GIR estimates.

**Conclusions:**

We demonstrate that clinical and research biomarkers can be used to estimate tissue specific insulin sensitivity in adults with Type 1 diabetes.

## 1 INTRODUCTION

Despite the fact that impaired insulin sensitivity is associated with increased risk of cardiovascular disease, microvascular complications, severe hypoglycaemia, diabetic ketoacidosis and premature death, it is frequently overlooked in people with Type 1 diabetes.^1^ Unlike Type 2 diabetes, in which insulin resistance is a predominant feature,^2^ the challenge in recognising and quantifying insulin resistance in Type 1 diabetes stems from its coexistence with absolute insulin deficiency. This duality complicates the assessment of metabolic health and the identification of individuals at increased risk for Type 1 diabetes complications.

The euglycaemic hyperinsulinaemic clamp remains the ‘gold standard’ for assessing insulin sensitivity, offering precision by directly measuring the body's metabolic response to a fixed insulin dose.^3^ However, this method is resource‐intensive and time‐consuming and is therefore restricted to research settings and clinical trials, rather than being part of routine clinical management in Type 1 diabetes. Enhancing its utility, the inclusion of stable isotope‐labelled glucose infusion during the clamp provides additional granularity.^3^, ^4^, ^5^ By differentiating endogenous glucose production (EGP), which is generated mainly by the liver and, to a lesser extent, the kidneys, from peripheral glucose utilisation by muscle and adipose tissue (reflected by the exogenous glucose‐infusion rate [GIR]), the stable‐isotope approach offers a comprehensive appraisal of hepatic output and peripheral uptake when the insulin infusion rate is high enough to suppress most hepatic glucose release. Reduced EGP suppression and reduced GIR during the clamp are distinctive markers of liver and muscle insulin resistance, respectively.^6^

Despite these advancements, measuring these variables in clinical practice has remained a challenge. To bridge this gap, we have developed predictive formulae for EGP and GIR in individuals with Type 1 diabetes to estimate hepatic and peripheral insulin sensitivity, respectively. These formulae, using clinically available biomarkers, offer a practical approach to identifying tissue specific insulin resistance in people with Type 1 diabetes. We developed an online calculator (accessible at: https://bit.ly/EGP-GIR-calculator), allowing clinicians and researchers to predict the existence of hepatic and peripheral tissue insulin resistance. By simplifying the estimation of hepatic and peripheral insulin sensitivity, we aim to enhance the understanding of metabolic risk in Type 1 diabetes and support targeted interventions to mitigate complication risk.

## 2 METHODS

We conducted two‐stage euglycaemic hyperinsulinaemic clamps in 40 adults with Type 1 diabetes enrolled in the INTIMET trial (Insulin resistance in Type 1 diabetes managed with metformin, ACTRN12619001440112).^7^, ^8^ As previously published, the initial stage was a 2‐h deuterated glucose tracer equilibration period (6,6‐^2^H_2_‐glucose, Cambridge Isotope Laboratories, priming bolus 5 mg/kg; administered over 5 min then continuous infusion 0.05 mg/kg/min), and a variable insulin infusion (Actrapid, Novo Nordisk) in participants with Type 1 diabetes to achieve a target glucose of 5.5 mmol/L.^7^ During the low‐dose and high‐dose phases, a fixed insulin infusion (20 and 60 mU/m^2^/min, respectively; dosed by body surface area) was administered for 2 h, and a dextrose infusion (25% dextrose with 2% 6,6‐^2^H_2_‐glucose enrichment) was used to maintain euglycaemia and stable tracer enrichment. Hepatic insulin sensitivity was defined by EGP after partial suppression during the steady state of the low‐dose stage of the clamp calculated using the Powrie et al. formula,^9^ and peripheral insulin sensitivity by the GIR during the high‐dose stage required to maintain the target glucose during steady state. We used positive chemical ionisation gas chromatography–mass spectrometry with the glucose methyloxime pentapropionate derivatisation strategy to measure glucose tracer enrichment, per published methods.^10^ EGP and GIR were determined using data from the last 30 min of the clamp (steady state with target glucose 5.0–5.5 mmol/L) and were corrected for fat‐free mass (FFM).

Participants were characterised with 33 demographic, clinical and research biomarkers (listed in Table 1). All available biomarkers were offered to a logistic regression exhaustive search to derive equations predicting ‘unfavourable EGP and GIR outcomes’—EGP below (good) (coded as 0)/above (bad) (coded as 1) 6.18 μM/kg FFM/min and GIR above (good) (coded as 0)/below (bad) (coded as 1) 60.42 μM/kg FFM/min. The cut‐offs were determined as the median value of EGP and GIR. Based on the number of study subjects, we limited the models to a maximum of four variables. The best models for prediction of unfavourable EGP and GIR were selected based on the lowest Akaike Information Criterion, and models' significance was assessed using loglikelihood. Correlations between EGP or GIR and clinical and research parameters were measured using Spearman *ρ* coefficient. Significance was set at *p* < 0.05.

**TABLE 1:** General characteristics of study participants with Type 1 diabetes.

Variable	Mean ± SD or median (LQ, UQ)
Age (years)	38 ± 9
BMI (kg/m^2^)	26.4 ± 3.8
WHR	0.89 ± 0.08
WHtR	0.51 ± 0.06
PP (mmHg)	45.8 ± 8.2
MAP (mmHg)	88.2 ± 8.6
Type 1 diabetes duration (years)	23 ± 9
HbA1c (%)	7.5 ± 0.9
HbA1c (mmol/mol)	58 ± 10
Total daily insulin dose (IU)	46 (36, 60)
TC (mM)	4.3 ± 0.8
TG (mM)	0.8 (0.6, 1.1)
HDL‐C (mM)	1.2 (1.1, 1.6)
LDL‐C (mM)	2.6 ± 0.6
TC/HDL‐C	3.3 ± 0.7
TG/HDL‐C	0.7 (0.4, 1.0)
HDL‐C/LDL‐C	0.5 (0.4, 0.7)
Non‐HDL‐C	2.9 ± 0.7
Albumin (g/L)	38 (36, 39)
Total protein (g/L)	64.0 ± 3.5
Total globulin (g/L)	26.8 ± 3.2
ALT (U/L)	24 (16, 31)
AST (U/L)	18 (13, 23)
GGT (U/L)	15 (12, 29)
Uric acid (μM)	0.25 ± 0.07
Bilirubin (μM)	9 (8, 14)
IGF‐1 (nM)	18.9 ± 4.7
ICAM (ng/mL)	213 ± 48
eSelectin (ng/mL)	35.0 ± 14.2
Adiponectin (μg/mL)	4.9 (3.2, 10.1)
IL‐6 (pg/mL)	1.0 (0.7, 1.5)
CRP (mg/L)	2.8 (2.8, 2.8)
ALP (U/L)	65 (50, 92)
SHBG (nM)	54 (37, 77)
EGP (μmol/kg FFM/min)	6.1 ± 2.0
GIR (μmol/kg FFM/min)	61.4 ± 20.0

Abbreviations: ALP, alkaline phosphatase; ALT, alanine aminotransferase; AST, aspartate aminotransferase; BMI, body mass index; CRP, C‐reactive protein; EGP, endogenous glucose production; GGT, gamma‐glutamyl transpeptidase; GIR, glucose infusion rate; HDL‐C/LDL‐C, high‐density/low‐density lipoprotein cholesterol ratio; IGF‐1, insulin‐like growth factor 1; IL, interleukin; MAP, mean arterial pressure; PP, pulse pressure; SHBG, sex hormone binding globulin; TC, total cholesterol; TG, triglycerides; WHR, waist to hip ratio; WHtR, waist to height ratio.

## 3 RESULTS

Participant characteristics are shown in Table 1. Tracer‐derived EGP and GIR ranged from 1.29 to 9.82 and 20.35 to 100.83 μmol/kg FFM/min, respectively (corresponding to 0.14–1.29 and 2.42–11.61 mg/kg/min), within the mean ranges reported in published hyperinsulinaemic clamp studies in adults with Type 1 diabetes.^11^

Table 2 shows univariate (Spearman) correlations between selected measured parameters and EGP and GIR. The only statistically significant correlation of EGP was with alkaline phosphatase (ALP). GIR correlated with BMI, HbA1c, lipids, and inflammatory biomarkers. The correlation between EGP and GIR was non‐significant (*r* = −0.19, *p* = 0.22).

**TABLE 2:** Selected univariate correlation (Spearman *ρ* and *p*‐value) of EGP and GIR.

Variables	EGP	*p*‐value	GIR	*p*‐value
BMI	0.187	0.248	−0.352	**0.026**
WHR	−0.043	0.791	−0.395	**0.012**
WHtR	0.195	0.228	−0.452	**0.003**
HbA1c	0.177	0.274	−0.342	**0.031**
Total daily insulin dose	0.302	0.065	−0.609	**<0.0001**
IGF‐1	0.129	0.428	0.017	0.919
LDL‐C	−0.030	0.853	−0.354	**0.025**
TG	0.054	0.741	−0.208	0.198
TC/HDL‐C	0.049	0.762	−0.472	**0.002**
HDL‐C/LDL‐C	0.023	0.887	−0.449	**0.004**
Non‐HDL‐C	0.030	0.852	−0.369	**0.019**
ALT	−0.088	0.590	−0.407	**0.009**
AST	0.025	0.878	−0.301	0.059
GGT	0.072	0.658	−0.433	**0.005**
sICAM	0.049	0.762	−0.388	**0.013**
seSelectin	0.300	0.062	−0.335	**0.035**
Adiponectin	0.102	0.529	0.372	**0.018**
CRP	0.108	0.508	−0.360	**0.022**
ALP	0.323	**0.042**	−0.221	0.169
Uric acid	−0.208	0.198	−0.134	0.409

Abbreviations: ALP, alkaline phosphatase; ALT, alanine aminotransferase; AST, aspartate aminotransferase; BMI, body mass index; CRP, C‐reactive protein; EGP, endogenous glucose production; GGT, gamma‐glutamyl transpeptidase; GIR, glucose infusion rate; HDL‐C/LDL‐C, high‐density/low‐density lipoprotein cholesterol ratio; IGF‐1, insulin‐like growth factor 1; IL, interleukin; MAP, mean arterial pressure; PP, pulse pressure; SHBG, sex hormone binding globulin; TC, total cholesterol; TG, triglycerides; WHR, waist to hip ratio; WHtR, waist to height ratio.

**FIGURE 1:**
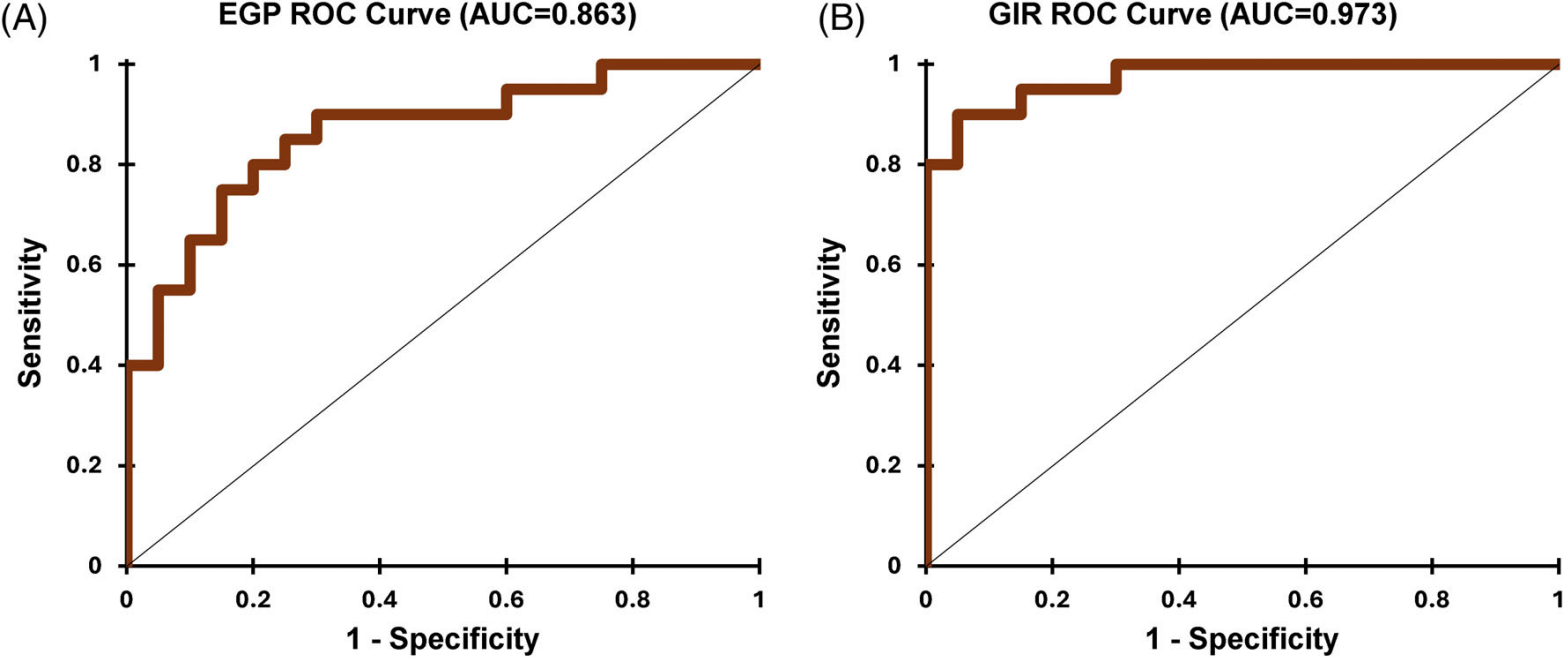
Receiver operating characteristic (ROC) curves for the best logistic regression models for detection of unfavourable: (A) endogenous glucose production (EGP) and (B) glucose infusion rate (GIR).

The best model to determine an unfavourable GIR contained plasma triglycerides (TG, *p* = 0.03), total daily insulin dose (TDI, *p* = 0.01), insulin‐like growth factor 1 (IGF‐1, *p* = 0.04) and aspartate aminotransferase (AST, *p* = 0.05) measurements. The model pseudo‐*R* ^2^ (Nagelkerke) was 0.83 (*p* < 0.0001, goodness of fit Hosmer–Lemeshow test *χ* ^2^ = 2.29, *p* = 0.97). Model sensitivity was 90%, specificity—95%, overall accuracy—92.5% and area under the receiver operating characteristic curve (AUROC) = 0.97, with only 3 of 40 observations (7.5%) misclassified (Figure 1B). The formula for probability of an unfavourable (below median) GIR:

$$\text{Logit}\left(\mathrm{GIR}\right)=\begin{array}{l} –80.49-7.36\times \mathrm{Ln}\left(\mathrm{TG}\left[\mathrm{mM}\right]\right)+18.92\times \mathrm{Ln}\left(\mathrm{TDI}\left[\mathrm{IU}\right]\right)\\ -\;0.62\times \text{IGF1}\left[\mathrm{nM}\right]+6.09\times \mathrm{Ln}\left(\mathrm{AST}\left[U/L\right]\right). \end{array}$$

In fivefold cross‐validation (with 100 repeats) the overall accuracy was 81%, with sensitivity 83% and specificity 79% and AUROC = 0.90. Bootstrapped (*n* = 1000) optimism corrected AUROC was 0.95.^12^, ^13^ We also explored an alternative, next‐best model identified through exhaustive search when IGF‐1 was excluded. In this model, IGF‐1 was replaced by HbA1c, resulting in attenuated performance compared with the full specification (pseudo‐*R* ^2^ = 0.74; AUROC = 0.96; overall accuracy = 85%), as detailed in the Supporting Information.

The best model to determine an unfavourable EGP (indicative of hepatic insulin resistance) contained TG (*p* = 0.06), TDI (*p* = 0.17), ALP (*p* = 0.09), and uric acid (*p* = 0.004) measurements. The model pseudo‐*R* ^2^ (Nagelkerke) was 0.50 (*p* = 0.001, Goodness of fit Hosmer–Lemeshow test *χ* ^2^ = 5.55, *p* = 0.70). Model sensitivity, specificity and overall accuracy were each 80% and AUROC = 0.86, with only eight (of 40, 20%) observations misclassified (Figure 1A). The formula for probability of unfavourable (above median) EGP was:

$$\text{Logit}\left(\mathrm{EGP}\right)=\begin{array}{l} –27.51-2.63\times \mathrm{Ln}\left(\mathrm{TG}\left[\mathrm{mM}\right]\right)+2.25\times \mathrm{Ln}\left(\mathrm{TDI}\left[\mathrm{IU}\right]\right)+2.26\\ \times \mathrm{Ln}\left(\mathrm{ALP}\left[U/L\right]\right)-6.13\times \mathrm{Ln}\left(\text{uric acid}\left[\mathrm{\mu M}\right]\right). \end{array}$$

In fivefold cross‐validation (with 100 repeats) the overall accuracy was 70%, with sensitivity 72% and specificity 68% and AUROC = 0.80. Bootstrapped (*n* = 1000) optimism corrected AUROC was 0.81.^12^, ^13^

## 4 DISCUSSION

Insulin resistance in people with Type 1 diabetes is associated with a higher risk of vascular and other complications.^14^, ^15^ Key insulin‐sensitive tissues include the liver, skeletal muscle, and adipose tissue, and it is ideal to consider each when assessing insulin sensitivity.^16^ Mathematically modelling glucose utilisation parameters in euglycaemic hyperinsulinaemic clamp studies is challenging. We have shown previously that various formulae for estimating glucose disposal rate (GDR) deliver different numerical results, but their interpretation (in terms of ‘high/low’ or ‘good/bad’ GDR) is remarkably consistent.^17^, ^18^ In this study we modelled the probability that a participant's EGP would exceed, and their GIR would fall below, the respective cohort medians (6.18 μmol/kg FFM/min for EGP and 60.4 μmol/kg FFM/min for GIR). Dichotomising at these empirically derived cut‐points separates individuals into relative ‘unfavourable’ versus ‘favourable’ phenotypes within the study population, allowing the output to be expressed as an intuitive probability rather than an abstract continuous value, without implying a definitive clinical threshold. Although mathematical formulae for EGP diurnal dynamics^19^ and EGP suppression during the clamp^16^ exist, we are not aware of any available for people with Type 1 diabetes.

Exhaustive logistic‐regression search selected six of 33 candidate variables. Interestingly, TG and TDI each predicted both unfavourable EGP and GIR. Higher TDI predicted higher odds of unfavourable EGP and lower GIR despite the weak negative correlation between EGP and GIR. Surprisingly, higher TG was linked to lower odds of unfavourable EGP and to higher GIR. All participants were normolipidemic (fasting TG ≤2.0 mmol/L, only two with fasting TG >1.7 mmol/L), and 25% were taking an HMG Co‐A reductase inhibitor, so these associations reflect variation within the laboratory reference range in the pre‐clamp fasting state rather than overt dyslipidaemia. Within that physiological range, incremental rises in TG were linked to lower EGP and higher GIR. Although the assay quantifies circulating (mainly VLDL‐bound) TG, these levels closely track the turnover of adipose‐stored TG, which acts as a metabolic buffer by releasing free fatty acids (FFAs) in the post‐absorptive state.^20^ FFA oxidation thereby conserves glucose for obligate glucose‐using tissues.

In people with Type 1 diabetes, the normal portal‐to‐systemic insulin gradient is lost because subcutaneous insulin first enters the systemic circulation; arterial and portal insulin concentrations therefore rise in parallel. Clamp‐tracer studies show that hepatic glucose output is already suppressed at modest systemic insulin levels, whereas considerably higher levels are needed to curb VLDL‐TG export.^21^ This earlier inhibition of EGP is reinforced by insulin‐mediated reductions in glucagon and circulating FFAs.^22^ Consequently, a fasting VLDL‐TG concentration toward the upper end of the reference range may mark individuals whose livers are insulin‐responsive even while VLDL secretion persists. By contrast, a higher TDI, an accepted proxy for whole‐body insulin resistance in T1D,^21^ predicted higher EGP and lower GIR.

We demonstrate a correlation between adiponectin and GIR. Adiponectin enhances insulin sensitivity by activating AMP‐activated protein kinase, which suppresses hepatic gluconeogenesis, reducing EGP and stimulating glucose uptake and oxidation in skeletal muscle. We and others showed that adiponectin is positively correlated with directly measured whole body GDR in euglycaemic hyperinsulinaemic clamps in people with Type 1 diabetes.^23^, ^24^

Uric acid had a counterintuitive effect and was associated with lower EGP in the liver. In normouraemic adults (♂ 0.24–0.51 mmol/L, ♀ 0.16–0.43 mmol/L) with Type 1 diabetes (the highest uric acid level in this group was 0.4 mmol/L), uric acid may reduce EGP in the liver by mitigating oxidative stress,^25^ which in turn reduces gluconeogenesis by inhibiting stress‐related signalling pathways like the c‐Jun N‐terminal kinase and Nuclear Factor kappa‐light‐chain‐enhancer of activated B cells pathways.^26^

An increase in IGF‐1 can be associated with an increase of peripheral glucose utilisation in muscles and adipose tissue through a combination of synergistic action with insulin, via direct activation of the IGF‐1 receptor^27^ and improved mitochondrial function.^28^ Its ability to enhance both insulin‐dependent and insulin‐independent pathways makes it a key player in promoting glucose uptake in muscles and adipose tissue. It has been shown that administration of IGF‐1 improves insulin sensitivity in people with Type 1 diabetes.^29^

Regarding the association between ALP and EGP, previous studies show a correlation between ALP and FGF‐23,^30^ which in turn showed a positive correlation with insulin resistance (albeit in people with diabetes complicated with chronic kidney disease).^31^, ^32^ ALP also has anti‐inflammatory properties, with its anti‐inflammatory actions potentially enhancing β‐oxidation^33^ and gluconeogenesis in hepatocytes.^34^ Similarly, increases in AST levels may indicate muscle stress,^35^ which may reduce glucose uptake. Also, the negative effects of AST on peripheral glucose utilisation might be explained by muscle mitochondrial dysfunction observed in Type 1 diabetes.^36^ However, we did not observe any statistically significant correlation between AST and either EGP or GIR.

Our calculator also allows visualisation of discordant EGP and GIR, that is, when unfavourable EGP is accompanied by favourable GIR and vice versa. This highlights the complex nature of insulin sensitivity in Type 1 diabetes. Exogenous insulin is required to optimise glycaemia in Type 1 diabetes, but standard insulin modalities (whether using multiple daily injections or insulin pumps) bypass the liver, resulting in peripheral hyperinsulinaemia, which is thought to be a major factor driving insulin resistance.^37^ Due to reliance on exogenous insulin, standard insulin resistance prediction tools, such as the HOMA‐IR, are not valid in Type 1 diabetes, and novel Type 1 diabetes specific tools are required.

## 5 STRENGTHS AND LIMITATIONS

Study strengths include the detailed subject characterisation of adults with Type 1 diabetes enabling an extensive exploratory modelling approach. We also used the “hot‐GINF” (hot glucose infusate) method to assess hepatic and skeletal muscle insulin sensitivity, and we believe ours is the first insulin resistance calculator with muscle and liver specificity in people with Type 1 diabetes. Study limitations include the relatively small number of subjects, though it is similar in size to most other clamp studies. The participants were of overall good metabolic control, without untreated hyperlipidaemia and hyperuricaemia, though relatively normal lipid and uric acid levels are common in people with Type 1 diabetes.^38^ Thus, we can only speculate about the effect of factors such as hyperlipidaemia and/or hyperuricaemia on EGP and GIR estimation. Our calculator has entry TG and uric acid values truncated at the upper limit of the reference range to avoid extrapolation beyond the data‐supported domain of the model. Requirement for IGF‐1 measurement may limit applicability in routine clinical care; however, simplified alternative models using more commonly available biomarkers (e.g., HbA1c) have been explored and are described in the Supporting Information. Median‐based classification was used as a distribution‐anchored modelling strategy in the absence of established absolute thresholds for tracer‐derived EGP and GIR in Type 1 diabetes, and should be interpreted as reflecting relative phenotypes rather than definitive clinical cut‐points. Finally, the absence of an independent external validation cohort is an important limitation. Given the intensive nature of clamp studies with tracer methodology in adult Type 1 diabetes, suitable independent datasets remain limited. The present models are not intended to provide precise individual‐level estimates of tissue‐specific insulin sensitivity, but rather to identify biologically meaningful patterns and relative phenotypes in research and translational settings. Any potential clinical application, such as informing consideration of adjunctive insulin‐sensitising approaches, would require prospective validation and should be regarded as exploratory.

## 6 CONCLUSIONS

Tissue specific estimation of insulin sensitivity appears to be feasible in people with Type 1 diabetes using readily available biomarkers. Accordingly, our findings should be interpreted as demonstrating feasibility and biological signal, rather than precise quantification of tissue‐specific insulin sensitivity at the individual level. In future, incorporating estimates of hepatic and muscle insulin sensitivity in clinical trials and ultimately, if sufficiently validated, in clinical practice, may help predict response to insulin sensitising lifestyle and pharmacological interventions. As a decision‐support tool, our online calculator has the potential to increase awareness of insulin resistance in adults with Type 1 diabetes and to support hypothesis generation and risk stratification, rather than to replace direct metabolic assessment. Validation studies are merited.

## AUTHOR CONTRIBUTIONS

**ASJ:** Design; conduct/data collection; analysis; writing manuscript. **JRS:** Conduct/data collection; writing manuscript. **GMK:** Conduct/data collection; writing manuscript. **CRB:** Conduct/data collection; writing manuscript. **DJH‐W:** Conduct/data collection; writing manuscript. **AJJ:** Writing manuscript. **JRG:** Design; conduct/data collection; writing manuscript.

## FUNDING INFORMATION

This study was supported by grants awarded by the Diabetes Australia Research Program (Type 1 Diabetes Millennium Award 2019) and St Vincent's Clinic Foundation. No funding body played any role in the study design and will not play a part in data collection, study analysis or manuscript preparation. JRS is supported by an Australian Government Research Training Program Scholarship, National Health and Medical Research Council (NHMRC) Post‐Graduate Scholarship and Diabetes Australia Post Graduate Award.

## CONFLICT OF INTEREST STATEMENT

The authors declare no conflicts of interest.

## Supporting information


**Data S1.** Supporting Information.

## Data Availability

The data that support the findings of this study are available on request from the corresponding author. The data are not publicly available due to privacy or ethical restrictions.
