# Supplementary material for: Interactive online calculator for estimation of muscle and hepatic insulin sensitivity in adults with Type 1 diabetes using clinical and research biomarkers
Source: Diabetes Obes Metab. 2026 Feb 2;28(4):3238–44. doi: 10.1111/dom.70517 (PMC12992206; doi:10.1111/dom.70517)
Supplement: Supplementary file 1 — Data S1. Supporting Information. [file DOM-28-3238-s001.docx]

**Supplementary material**

**Alternative GIR model excluding IGF-1**

An alternative, next-best model for estimation of unfavourable GIR was identified through exhaustive search after exclusion of IGF-1.

The model included plasma triglycerides (p = 0.037), total daily insulin dose (TDI; p = 0.022), HbA1c (p = 0.047), and AST (p = 0.042). The model demonstrated good performance, with a Nagelkerke pseudo-R² of 0.74 (p < 0.0001; Hosmer–Lemeshow goodness-of-fit χ² = 2.69, p = 0.95). Sensitivity, specificity, and overall accuracy were each 85%, with an AUROC of 0.96 and six of 40 observations (15%) misclassified.

The formula for the probability of an unfavourable (below-median) GIR was:

Logit(GIR) = −105.97 − 6.62 x Ln(TG [mmol/L]) + 15.27 x Ln(TDI [IU]) + 2.58 x HbA1c [%] + 9.16 x Ln(AST [U/L]).
